# Association Between Lithium Use and Periprosthetic Fracture After Total Hip Arthroplasty

**DOI:** 10.1016/j.artd.2025.101851

**Published:** 2025-09-26

**Authors:** Rafa Rahman, Billy Kim, Benjamin Basseri, Michael Mazzucco, Alexander McLawhorn

**Affiliations:** Department of Orthopedic Surgery, Hospital for Special Surgery, New York, NY, USA

**Keywords:** Periprosthetic fracture, Osteoporosis, Total hip arthroplasty, Lithium, Bone mineral density

## Abstract

**Background:**

Periprosthetic fracture (PPFx) is a devastating complication following total hip arthroplasty (THA), with concern for higher risk in osteoporotic patients. Lithium is associated with higher bone mineral density, and has emerged as a potential low-cost, widely available method for preventing fractures, promoting fracture healing, and improving implant osseointegration. This study investigated the association between lithium use and risk of PPFx following THA.

**Methods:**

Retrospective review of the PearlDiver Mariner Patient Claims Database was performed, querying all patients who underwent THA for osteoarthritis from 2010 to 2022. Lithium-use patients were those who filled lithium prescriptions for at least 90 days before and 90 days after THA. These patients were propensity-score matched to controls not on lithium in a 1:4 ratio, matching for age, diagnosis of bipolar disorder, gender, body mass index, Charlson Comorbidity Index, and insurance. PPFx rate was compared between groups at 90 days and 2 years postoperatively. Secondarily, rate of aseptic loosening, revision, and prosthetic joint infection were compared between groups.

**Results:**

Four thousand six hundred seventy patients were included, with 934 patients on lithium and 3736 controls. There was no difference in PPFx rate (90 day: lithium 1.3% vs no lithium 1.2%, *P* = .97; 2 year: lithium 1.7% vs no lithium 1.9%, *P* = .93), aseptic loosening, revision, or prosthetic joint infection.

**Conclusions:**

Despite the theoretical benefit of lithium on bone density, it was not associated with a difference in the rate of PPFx or other surgical complication following THA. Further work is needed to address treatment of osteoporosis and prevention of periprosthetic fracture in the arthroplasty population.

## Introduction

Periprosthetic fractures (PPFx) can be devastating complications for patients following total hip arthroplasty (THA), often requiring surgical management [1]. There can be substantial morbidity and mortality following this complication, with one study estimating mortality in the first month after PPFx at 9.3%, and in the first year at 22.3% [2]. Over half of patients who suffer PPFx may not regain the same walking status they had prior to the fracture [2].

Recent reviews have highlighted the increased risk of PPFx in patients with osteoporosis and have called for optimization of bone health prior to arthroplasty [3,4]. Other studies have identified higher risk of PPFx among female and elderly patients, and it is known that women over 65 years are among the highest risk for osteoporosis [5,6]. Aseptic loosening, another complication of THA, has also been found to be associated with poor bone quality [5].

Although the importance of optimizing bone mineral density (BMD) prior to arthroplasty seems apparent, osteoporosis remains undertreated in this population. Few arthroplasty patients undergo dual-energy X-ray absorptiometry (DXA) scans to evaluate BMD, and pharmacotherapy for osteoporosis remains under-prescribed among arthroplasty patients [7,8]. While osteoporosis management prior to the index arthroplasty is important, treatment of osteoporosis is also necessary following PPFx, should it occur. Worse outcomes have been shown following PPFx after hip arthroplasty in patients with more severe BMD loss [9]. Finite element analysis models of plating in the setting of PPFx after THA has demonstrated higher bone strain and peak plate stress in osteoporotic vs healthy femurs [10].

The side effects and cost of medications for osteoporosis leave room for improvement in strategies to optimize bone health. Bisphosphonates and denosumab carry the rare but devastating risk of osteonecrosis of the jaw, among other side effects [[11], [12], [13]]. Newer formulations of bisphosphonates and newer agents are available, but can come at a higher price for patients, based on publicly available drug cost estimation tools [14,15]. Analysis of osteoporosis medications in the prevention of PPFx following arthroplasty for femoral neck fractures has found that intravenous bisphosphonates and newer anti-osteoporotic agents would need substantial cost reductions in order to be cost effective as prophylaxis against PPFx [16]. Some patients also prefer tablets, impacting the mode of administration for anti-osteoporotic medication [17].

Lithium presents a possible alternative method to improve bone health. It has been associated with higher BMD and has emerged as a potential low-cost and widely available method for fracture healing [18,19]. Experimental evidence from animal models have found that lithium improves osteoporotic fracture healing and improves implant osseointegration and fixation [[20], [21], [22]]. Outside of animal models, a meta-analysis found lithium to be associated with a 20% decreased risk of fracture [23], and another study found that lithium decreased the risk of all fractures as well as specifically Colles’ fractures and spine fractures [24]. The Lithium for Fracture Treatment (LiFT) randomized control trial is currently seeking to assess efficacy of lithium in long bone fracture healing [25].

The role of lithium in preventing PPFx after arthroplasty has not been studied. Thus, the primary goal of this study was to determine whether there is an association between perioperative use of lithium in patients undergoing THA and rate of PPFx. We hypothesized that patients taking lithium perioperatively would have a lower rate of PPFx following THA. Secondarily, this study compared rate of aseptic loosening, revision surgery, prosthetic joint infection (PJI), and medical complications among those taking lithium perioperatively vs those who were not. Among patients with PPFx, we also compared the rate of nonunion or delayed union.

## Material and methods

The PearlDiver Mariner Patient Claims Database (PearlDiver Technologies, Colorado Springs, CO) was queried for all patients who underwent primary THA for hip osteoarthritis from January 2010 to October 2022. This administrative claims database is a Health Insurance Portability and Accountability Act compliant, deidentified database that includes claims across all payer types (commercial insurance, Medicare, Medicaid, etc.) for 165 million patients and has been validated for use in other similar arthroplasty studies [26,27]. All queries were performed using pre-established outcome variables within the PearlDiver software, Current Procedural Terminology, or International Classification of Diseases (ICD-9 and ICD-10) codes outlined in Supplementary Table A.1. In compliance with Health Insurance Portability and Accountability Act, counts are reported in the database as <11 if there were less than or equal to 10 occurrences.

Patients were stratified into lithium use (treatment) and non-use (control) groups based on filling of prescriptions of lithium medications for a duration of at least 90 days before and 90 days after the date of THA, within a 120-day window before and 120-day window after date of surgery. Pharmacy claims for prescriptions filled before and after discharge were captured in this database. Medication administration was assumed based on filling of prescriptions. Lithium medications included all compounds (lithium carbonate, lithium citrate, and lithium aspartate) and formulations (pills, capsules, liquid, immediate and extended release) (Supplementary Table A.1). Patients with end-stage renal disease were excluded to account for impaired renal clearance of lithium. Additionally, patients with diagnosis of osteoporosis or those taking bisphosphonate medications or other agents for osteoporosis treatment (denosumab, romosozumab, teriparatide, abaloparatide, raloxifene) were excluded.

After applying the exclusion criteria, there were 934 patients in the lithium use and 705,101 patients in the non-use group with a follow-up duration of at least 90 days after THA. Among patients with a follow-up duration of at least 2 years after THA, there were 755 patients in the lithium use and 551,739 in the non-use group. Control and treatment groups were propensity score matched at a 4:1 ratio for both 90-day and 2-year cohorts, and were matched for patient age, diagnosis of bipolar disorder, gender, obesity (body mass index ≥ 30 kg/m^2^), Charlson Comorbidity Index (CCI), and insurance type. Insurance was categorized as commercial, Medicare, Medicaid, and other. Propensity score matching included diagnosis of bipolar disorder, as lithium is commonly used in treatment of the disease [28]. Diagnosis of bipolar disorder was based on ICD-9 and ICD-10 codes (Supplementary Table A.1). Matching was performed using the nearest neighbor method and a standard caliper of 0.1. Patient characteristics were compared between control and treatment groups after matching. Rates of 90-day and 2-year postoperative complications were compared between groups.

Patient and surgical data variables included patient age, gender, CCI, diagnosis of obesity, region, year of surgery, insurance type, and hospital length of stay. Postoperative 90-day outcomes using pre-encoded complications in the PearlDiver software included readmissions, acute kidney injury, cardiac arrest, deep vein thrombosis, wound dehiscence, hematoma, nerve injury, pneumonia, pulmonary embolism, transfusion, and urinary tract infection. Surgical complications were assessed at 90 days and 2 years using the appropriate cohorts, which included PPFx, aseptic loosening, revision of any component, and PJI. A separate subgroup analysis was performed for patients who suffered PPFx, comparing rate of nonunion and delayed union, which were assessed based on ICD-9 and ICD-10 codes (Supplementary Table A.1), at 90 days and 2 years after THA among those on lithium vs those not on lithium.

### Data analyses

Statistical analyses were performed using R (R Foundation, Vienna, Austria) functions built within the Pearldiver Bellweather software. After propensity score matching, univariable analyses were performed to compare means and rates of patient variables and complications between control and treatment groups. Univariable tests included one-way analysis of variance and Chi-Square test as appropriate. *P* values <0.05 were considered statistically significant.

### Sensitivity analysis

Additional sensitivity analysis was performed to further stratify bipolar disorder by severity. A similar 4:1 ratio between control and treatment groups for propensity matching was performed, again matching for patient age, diagnosis of bipolar disorder, gender, obesity, CCI, and insurance type, and with the addition of bipolar disease severity. Severity was categorized as mild, moderate, or severe, based on ICD-9 and ICD-10 codes (Supplementary Table A.1). The same outcomes were then assessed, including postoperative 90-day medical complications and both 90-day and 2-year surgical complications.

This study was approved as exempt by our institutional review board (IRB #2023-2068).

## Results

Controlling for patient age, diagnosis of bipolar disorder, gender, obesity, preoperative comorbidity risk profile, and insurance, 4670 total patients (934 lithium-users, 3736 control) were included in the 90-day cohort and 3775 total patients (755 lithium users, 3020 control) were included in the 2-year cohort. Control and treatment groups had similar patient characteristics (Table 1). There was no difference in the primary outcome of PPFx between the control group not on lithium compared to those on lithium within 90 days or 2 years of surgery, nor in the secondary outcomes of aseptic loosening, revision surgery, or PJI (Table 2). There was a higher rate of readmission in the control group compared to the group on lithium (9.2% vs 6.4%, *P* = .008) and a higher rate of pneumonia (1.8% vs 0.5%) (Table 2).

**Table 1:** Comparison of THA patient characteristics by perioperative lithium use.

Patient characteristics	No lithium use (n = 3736)	Lithium use (n = 934)	*P* value
Age, mean (sd)	59.8 (9.8)	59.7 (10.2)	.86
Gender, n (%)			.88
Male	1523 (40.8%)	384 (41.1%)	
Female	2213 (59.2%)	550 (58.9%)	
CCI, mean (sd)	2.1 (1.9)	2.1 (2.1)	.61
Obesity (BMI ≥ 30kg/m^2^)	861 (23.0%)	222 (23.8%)	.67
Bipolar disorder, n (%)	3256 (87.2%)	814 (87.2%)	1.0
Region, n (%)			.32
Midwest	991 (26.5%)	235 (25.2%)	
Northeast	920 (24.6%)	251 (26.9%)	
South	1311 (35.1%)	307 (32.9%)	
West	501 (13.4%)	139 (14.9%)	
Unknown	13 (0.3%)	2 (0.2%)	
Insurance plan, n (%)			.86
Commercial	2435 (65.2%)	597 (63.9%)	
Medicare	1032 (27.6%)	270 (28.9%)	
Medicaid	206 (5.5%)	50 (5.4%)	
Other	63 (1.7%)	17 (1.8%)	
Hospital Length of Stay (d), mean (sd)	2.7 (2.1)	2.6 (1.5)	.48

BMI, body mass index; sd, standard deviation.

**Table 2:** Comparison of Medical and Surgical Complication Rates among THA Patients with No Lithium Use vs those on Lithium.^a^

90 D postoperative	Total (n = 4670)	No lithium use (n = 3736)	Lithium use (n = 934)	*P* value
Readmissions	404 (8.7%)	344 (9.2%)	60 (6.4%)	**.01**
Any medical complication	487 (10.4%)	404 (10.8%)	83 (8.9%)	.10
Acute kidney injury	87 (1.9%)	74 (2.0%)	13 (1.4%)	.29
Cardiac arrest	4 (0.1%)	4 (0.1%)	0 (0.0%)	.71
Deep vein thrombosis	36 (0.8%)	29 (0.8%)	7 (0.7%)	1.00
Wound dehiscence	65 (1.4%)	55 (1.5%)	10 (1.1%)	.44
Hematoma	30 (0.6%)	26 (0.7%)	4 (0.4%)	.49
Nerve injury	0 (0.0%)	0 (0.0%)	0 (0.0%)	-
Pneumonia	71 (1.5%)	66 (1.8%)	5 (0.5%)	**.01**
Pulmonary embolism	8 (0.2%)	8 (0.2%)	0 (0.0%)	.33
Transfusion	110 (2.4%)	93 (2.5%)	17 (1.8%)	.28
Urinary tract infection	183 (3.9%)	148 (4.0%)	35 (3.7%)	.84
Periprosthetic fracture	57 (1.2%)	45 (1.2%)	12 (1.3%)	0.97
Aseptic loosening	15 (0.3%)	13 (0.3%)	2 (0.2%)	0.75
Revision surgery	86 (1.8%)	68 (1.8%)	18 (1.9%)	0.94
PJI	101 (2.2%)	88 (2.4%)	13 (1.4%)	0.09

2 Y postoperative	Total (n = 3775)	No lithium use (n = 3020)	Lithium use (n = 755)	*P* value
Periprosthetic fracture	69 (1.8%)	56 (1.9%)	13 (1.7%)	.93
Aseptic loosening	52 (1.4%)	41 (1.4%)	11 (1.5%)	.97
Revision surgery	141 (3.7%)	111 (3.7%)	30 (4.0%)	.78
PJI	124 (3.3%)	104 (3.4%)	20 (2.6%)	.33

Bold values indicate *P* values which meet the significance threshold of α = 0.05.

a Propensity-score matched between nonlithium users and lithium-use patients in 4:1 ratio based on age, diagnosis of bipolar disorder, gender, CCI, body mass index, and insurance type.

Among patients with PPFx, there were no patients either on lithium (n = 13) or not on lithium (n = 56) who suffered delayed union or nonunion of the PPFx at 90 days or 2 years.

### Sensitivity analysis

Sensitivity analysis, which further matched patients based on bipolar disease severity, in addition to the variables matched for in the primary analysis, found no difference in surgical complications including PPFx, aseptic loosening, revision surgery, and PJI at 90 days or 2 years postoperatively (Supplementary Table A.2). There was again a higher rate of readmission in the control group compared to the group on lithium (9.3% vs 6.4%, *P* < .01) and a higher rate of pneumonia (1.5% vs <1.2%, *P* = .04). These findings are all consistent with those of the primary analysis.

## Discussion

This study sought to determine whether perioperative lithium use was associated with the risk of PPFx and other medical and surgical complications following THA. We found that there was no difference in the rate of PPFx, aseptic loosening, revision surgery, or PJI among those on lithium compared to those not on lithium at 90 days or 2 years after surgery. Among patients who sustained a PPFx, there was no difference in rate of nonunion between patients on lithium vs those who were not.

These results were contrary to our hypothesis, and an unexpected finding given studies demonstrating higher BMD in patients on lithium [18], as well as animal model studies demonstrating improved fracture healing, bone formation, and osseointegration of implants with lithium use [[20], [21], [22]]. One explanation may be the role that bipolar disorder plays in modulating fracture risk. Lithium is commonly used in the treatment of bipolar disorder [28] and multiple studies have demonstrated that patients with bipolar disorder are at a higher risk of fracture overall [[29], [30], [31]]. However, there are also studies which suggest decreased risk of overall fracture in patients with bipolar disorder who are on lithium, compared to those who are not. Ng et al. found in a large United Kingdom cohort that patients with bipolar disorder on lithium had a lower risk of fracture that those on nonlithium treatments, including antipsychotics or mood-stabilizing antiepileptics [28]. In a similar vein, Lassas et al. found that patients with bipolar disorders who had higher BMD were more likely to be on lithium [32]. With regards to osteoporosis, lower BMD is seen in those with bipolar disorder [33]. Köhler-Forsberg et al. found that patients with bipolar disorder were more likely to have osteoporosis, but when treated with lithium, found that this risk decreased [34]. Importantly, our study corrects for confounding related to the diagnosis of bipolar disorder by including this in the propensity-match algorithm. However, while we control for the diagnosis, our data does not allow the granularity to determine if there are inherent differences in those patients with bipolar disorder who are on lithium vs those who are not. There may be differences in severity of the individual’s bipolar disorder which require lithium vs another treatment or no treatment at all. As evidenced by the previously mentioned studies, bipolar disorder in and of itself and lithium when used in the context of bipolar disorder can have varying effects on fracture risk.

We additionally found that at 90 days, patients not on lithium had a higher readmission rate and higher rate of pneumonia compared to patients on lithium. While these findings exhibited statistical significance, they do not suggest clinical significance given the small differences in the rates. Readmissions occurred at 9.2% in the control group vs 6.4% in the lithium group, and pneumonia occurred at 1.8% in the control group compared to 0.5% in the lithium group. Further supporting the likelihood that these differences are statistically but not clinically significant are the fact that the propensity-score matching algorithm controlled for age, obesity, and preoperative comorbidity risk profile. We found no reason to believe the control group was on average sicker than the lithium-use group.

While our current findings do not support the use of lithium in THA patients, it is possible that further work is needed to explore the nuances of lithium dosing and effects on BMD and risk reduction of complications following THA. Lithium upregulates pathways involved in osteoblastic mineralization by inhibiting glycogen synthase kinase-3β [35]. Experiments in rat models have found that lithium improves healing of osteoporotic femur fractures compared to placebo and increases bone formation in distraction osteogenesis for tibial osteotomies [20,21]. Separate studies in rat models have found lithium to improve osseointegration of tibial implants, implant fixation, and bone formation in osteoporotic rats [22]. Despite these successes, Bernick et al. and Vachhani et al.’s studies in rat models illustrated the need to optimize dosing, onset, and duration of lithium treatment to enhance healing [36,37]. Similar optimization may need to be made in clinical use of lithium for improving BMD and reducing fracture risk in order to see desired results. To our knowledge, such parameters for lithium administration for the purpose of bone health are not established.

Despite the promising role of lithium in decreasing fracture risk and trials seeking to assess the efficacy of lithium in fracture healing [[23], [24], [25]], our results have not borne out lithium as an effective tool to decrease PPFx risk in the THA population. Nonetheless, osteoporosis and efforts to reduce fracture risk remain a critical problem in the arthroplasty population. Holzer et al. underscore this issue in their study where, of 119 patients who met the criteria for DXA testing to evaluate BMD prior to THA or total knee arthroplasty, only 17.6% had DXA testing, and of those, 33% were found to have osteoporosis. Furthermore, only 11 of 49 patients who met the criteria for pharmacologic treatment of osteoporosis were prescribed pharmacotherapy in the 6 months before or after arthroplasty [7]. A separate study found that among patients who sustained PPFx after THA, 57% met the criteria that warranted osteoporosis treatment, but only 8% received treatment [8]. More research is needed to further explore lithium and alternative treatments in treating osteoporosis and reducing postoperative complications in this population.

This study is not without limitations. Our results are subject to the limitations associated with use of a large database, in this case, the PearlDiver Mariner Patient Claims Database. This includes missing data and potential issues with accuracy of data [38,39]. While we used a comprehensive claims database with a large patient sample, given the rarity of PPFx, we were limited in the number of matching criteria that could be incorporated in our propensity matching algorithm, and future studies may benefit from even larger patient samples. Nonetheless, we feel that the combination of our exclusion criteria and matching variables accounted for any major confounders that may have biased our results. Furthermore, in our sensitivity analysis, which did stratify by bipolar disorder severity, we found no difference in the surgical complications of interest at either 90 days or 2 years, and showed medical complication rates consistent with our primary analysis. Another limitation of using an administrative claims database is the level of granularity of the data. For example, while theoretically, matching criteria could have included history of fracture, given the wide range and heterogeneity of ICD codes for fracture-related diagnoses, this would not have provided a clinically interpretable nor practically feasible data point. While we could not control for history of fracture directly, we feel that by excluding patients who had a diagnosis of osteoporosis and those on medications traditionally used for treatment of osteoporosis, we indirectly control for confounding based on bone health and risk of fracture. The data we had access to also limited our ability to study medication adherence. As necessary with use of claims data, we made the assumption that patients were taking lithium based on their filling of prescriptions, although it is certainly possible that some patients may not have not consumed the medication. Due to inability to survey patients directly on their medication adherence and the lack of linking of the database to pharmacy re-filling data, we were unable to capture a more accurate description of medication adherence. Furthermore, we lack data on dosing and duration of lithium. Both of these factors may have impacted PPFx risk in a dose-response relationship, which could not be captured given the data available and due to our categorization of lithium use as a binary variable. Thus, any existing dose and duration-based associations with PPFx and other risks may have been lost in our analysis. Future studies would benefit from access to more granular data, where fracture history, lithium dosing and duration, and medication adherence could be better characterized. Inherent to our study is also the acknowledgment that the patients on lithium were on it due to bipolar disorder or another diagnosis, and unlikely for improvement of BMD. Future studies would benefit from prospectively studying the effect of lithium perioperatively in patients without bipolar disorder in a randomized trial. Such a study design would additionally allow an opportunity for measurement of BMD, comparing BMD prior to initiation of lithium to multiple time-points following its use. This analysis of the relationship between lithium and BMD could also be done in a non-arthroplasty population, with the goal being to more directly understand the effect of lithium on BMD in human subjects rather than trying to determine postoperative risks.

## Conclusions

While lithium has theoretical potential in decreasing fracture risk and improving fracture healing, among patients undergoing THA, there was no difference in the rates of PPFx or other surgical complications compared to controls. Given these findings, lithium does not appear to be an effective means of reducing these complications postoperatively. Further work is needed to address osteoporosis and prevention of PPFx and other complications in the arthroplasty population, with efforts to identify widely available, effective, and well-tolerated treatment strategies.

## CRediT authorship contribution statement

**Rafa Rahman:** Writing – review & editing, Writing – original draft, Visualization, Validation, Supervision, Resources, Project administration, Methodology, Investigation, Formal analysis, Data curation, Conceptualization. **Billy Kim:** Writing – review & editing, Methodology, Investigation, Formal analysis. **Benjamin Basseri:** Software, Resources, Methodology, Investigation, Conceptualization. **Michael Mazzucco:** Software, Methodology, Investigation. **Alexander McLawhorn:** Writing – review & editing, Supervision, Resources, Investigation, Conceptualization.

## Conflicts of interest

Rafa Rahman has Board member/committee appointments for American Society for Surgery of the Hand Clinical Research and Grantsmanship Committee.

Alexander McLawhorn receives Royalties from Globus Medical, Inc, - Consultant; Anticipated Royalties; other financial or material support from a company or supplier: HS2, LLC - Ownership Interest, HSS ASC Development Network - Ownership Interest, Joint Effort Administrative Services Organization, LLC - Ownership Interest, VITALIS- Ownership Interest.

The other authors declare no potential conflicts of interest.

For full disclosure statements refer to https://doi.org/10.1016/j.artd.2025.101851.

## Figures and Tables

**Table A.1:** ICD, Current Procedural Terminology, and Drug Codes

Diagnosis/Drug	PearlDiver CPT/ICD/Drug codes
THA	CPT-27130, ICD-10-P-0SRB019, ICD-10-P-0SRB01A, ICD-10-P-0SRB01Z, ICD-10-P-0SRB029, ICD-10-P-0SRB02A, ICD-10-P-0SRB02Z, ICD-10-P-0SRB039, ICD-10-P-0SRB03A, ICD-10-P-0SRB03Z, ICD-10-P-0SRB049, ICD-10-P-0SRB04A, ICD-10-P-0SRB04Z, ICD-10-P-0SRB069, ICD-10-P-0SRB06A, ICD-10-P-0SRB06Z, ICD-10-P-0SRB0J9, ICD-10-P-0SRB0JA, ICD-10-P-0SRB0JZ, ICD-10-P-0SRE009, ICD-10-P-0SRE00A, ICD-10-P-0SRE00Z, ICD-10-P-0SRE019, ICD-10-P-0SRE01A, ICD-10-P-0SRE01Z, ICD-10-P-0SRE039, ICD-10-P-0SRE03A, ICD-10-P-0SRE03Z, ICD-10-P-0SRE0J9, ICD-10-P-0SRE0JA, ICD-10-P-0SRE0JZ, ICD-10-P-0SR9019, ICD-10-P-0SR901A, ICD-10-P-0SR901Z, ICD-10-P-0SR9029, ICD-10-P-0SR902A, ICD-10-P-0SR902Z, ICD-10-P-0SR9039, ICD-10-P-0SR903A, ICD-10-P-0SR903Z, ICD-10-P-0SR9049, ICD-10-P-0SR904A, ICD-10-P-0SR904Z, ICD-10-P-0SR9069, ICD-10-P-0SR906A, ICD-10-P-0SR906Z, ICD-10-P-0SR90J9, ICD-10-P-0SR90JA, ICD-10-P-0SR90JZ, ICD-10-P-0SRA009, ICD-10-P-0SRA00A, ICD-10-P-0SRA00Z, ICD-10-P-0SRA019, ICD-10-P-0SRA01A, ICD-10-P-0SRA01Z, ICD-10-P-0SRA039, ICD-10-P-0SRA03A, ICD-10-P-0SRA03Z, ICD-10-P-0SRA0J9, ICD-10-P-0SRA0JA, ICD-10-P-0SRA0JZ, ICD-9-P-8151
Hip osteoarthritis	ICD-10-D-M160, ICD-10-D-M1610, ICD-10-D-M1611, ICD-10-D-M1612, ICD-10-D-M162, ICD-10-D-M1630, ICD-10-D-M1631, ICD-10-D-M1632, ICD-10-D-M164, ICD-10-D-M1650, ICD-10-D-M1651, ICD-10-D-M1652, ICD-10-D-M166, ICD-10-D-M167, ICD-10-D-M169, ICD-9-D-715.15
Bisphosphonate	NDC-00143964201, NDC-17478032705, NDC-45963044055, NDC-43598033011, NDC-47335096241, NDC-53150087101, NDC-63323096198, NDC-00078059061, NDC-42023015101, NDC-25021082682, NDC-23155017031, NDC-43598033111, NDC-67457039054, NDC-47335003540, NDC-42023016301, NDC-60505611000, NDC-25021080166, NDC-55111068507, NDC-00078038725, NDC-25021083082, NDC-23155018631, NDC-00078043561, NDC-55111068852, NDC-00004018682, NDC-00004019109, NDC-00378521553, NDC-00591377011, NDC-25021082761, NDC-42291033603, NDC-47781010333, NDC-54868532200, NDC-55111057503, NDC-60505279500, NDC-62756021840, NDC-67457052433, NDC-00006003144, NDC-00006071044, NDC-00093514001, NDC-00093514056, NDC-00093514101, NDC-00093514156, NDC-00093514256, NDC-00093517119, NDC-00093517120, NDC-00093517129, NDC-00093517144, NDC-00093517219, NDC-00093517220, NDC-00093517229, NDC-00093517244, NDC-00378356601, NDC-00378356701, NDC-00378356822, NDC-00378356893, NDC-00378356899, NDC-00378356922, NDC-00378356993, NDC-00378356999, NDC-00555071951, NDC-00555071954, NDC-00555072051, NDC-00555072054, NDC-00591003104, NDC-00591007704, NDC-00591317104, NDC-00591317304, NDC-16252059902, NDC-16252059944, NDC-16252060102, NDC-16252060144, NDC-16714063101, NDC-16714063102, NDC-16714063201, NDC-16714063202, NDC-16714063301, NDC-16714063302, NDC-21695090104, NDC-24658016273, NDC-24658016373, NDC-41616063583, NDC-41616063588, NDC-41616063683, NDC-41616063688, NDC-41616063768, NDC-41616063868, NDC-47335063768, NDC-47335063868, NDC-51079094101, NDC-51079094105, NDC-51079094201, NDC-51079094205, NDC-51224030110, NDC-54868385700, NDC-54868446200, NDC-54868548000, NDC-54868586000, NDC-54868586100, NDC-55111058801, NDC-55111058830, NDC-55111059048, NDC-55111059248, NDC-59746024202, NDC-59746024402, NDC-60429005572, NDC-60429005672, NDC-60505259201, NDC-60505259203, NDC-60505259301, NDC-60505259303, NDC-60505259404, NDC-60505259602, NDC-60505259604, NDC-60505259608, NDC-65862032730, NDC-65862032804, NDC-65862032904, NDC-67877024031, NDC-67877024131, NDC-68084032211, NDC-68084032294, NDC-76439013004, NDC-76439013104, NDC-00149047001, NDC-00149047101, NDC-00149047201, NDC-00149047801, NDC-00430047015, NDC-00430047115, NDC-00430047203, NDC-00430047801, NDC-00430097903, NDC-00591204403, NDC-00591207504, NDC-00591210230, NDC-00591210930, NDC-47335092860, NDC-54868438600, NDC-54868467100, NDC-54868606900, NDC-60505309702, NDC-00378328691, NDC-00378328891, NDC-15330009406, NDC-15330009506, NDC-00069010701, NDC-00069010901, NDC-00069018601, NDC-00517074501, NDC-00517074601, NDC-00703408551, NDC-00781314770, NDC-00781314784, NDC-00781314870, NDC-25021080210, NDC-25021080310, NDC-40042001910, NDC-55390012701, NDC-55390012901, NDC-55390015701, NDC-55390015901, NDC-55390020401, NDC-59923060110, NDC-59923060310, NDC-61703032418, NDC-61703032518, NDC-61703032618, NDC-63323073410, NDC-63323073510, NDC-67457043010, NDC-67457044610, NDC-67457061010
Other osteoporosis medications	GENERIC_DRUG-ROMOSOZUMAB-AQQG, GENERIC_DRUG-RALOXIFENE_HCL, GENERIC_DRUG-DENOSUMAB, GENERIC_DRUG-TERIPARATIDE, GENERIC_DRUG-ABALOPARATIDE
End-Stage Renal Disease	ICD-10-D-N186, ICD-9-D-5856, ICD-9-D-40301, ICD-9-D-40311, ICD-9-D-40391, ICD-9-D-40402, ICD-9-D-40403, ICD-9-D-40412, ICD-9-D-40413, ICD-9-D-40492, ICD-9-D-40493, ICD-10-D-I120, ICD-10-D-I1311, ICD-10-D-I132
Osteoporosis	ICD-10-D-M810, ICD-9-D-73300, ICD-9-D-73301, ICD-9-D-73302, ICD-9-D-73303, ICD-9-D-73309, ICD-10-D-M818, ICD-10-D-M8000XA, ICD-10-D-M8000XD, ICD-10-D-M8000XG, ICD-10-D-M8000XK, ICD-10-D-M8000XP, ICD-10-D-M8000XS, ICD-10-D-M80011A, ICD-10-D-M80011D, ICD-10-D-M80011G, ICD-10-D-M80011K, ICD-10-D-M80011P, ICD-10-D-M80011S, ICD-10-D-M80012A, ICD-10-D-M80012D, ICD-10-D-M80012G, ICD-10-D-M80012K, ICD-10-D-M80012P, ICD-10-D-M80012S, ICD-10-D-M80019A, ICD-10-D-M80019D, ICD-10-D-M80019G, ICD-10-D-M80019K, ICD-10-D-M80019P, ICD-10-D-M80019S, ICD-10-D-M80021A, ICD-10-D-M80021D, ICD-10-D-M80021G, ICD-10-D-M80021K, ICD-10-D-M80071S, ICD-10-D-M80021P, ICD-10-D-M80021S, ICD-10-D-M80022A, ICD-10-D-M80022D, ICD-10-D-M80022G, ICD-10-D-M80022K, ICD-10-D-M80022P, ICD-10-D-M80022S, ICD-10-D-M80029A, ICD-10-D-M80029D, ICD-10-D-M80029G, ICD-10-D-M80029K, ICD-10-D-M80029P, ICD-10-D-M80029S, ICD-10-D-M80031A, ICD-10-D-M80031D, ICD-10-D-M80031G, ICD-10-D-M80031K, ICD-10-D-M80031P, ICD-10-D-M80031S, ICD-10-D-M80032A, ICD-10-D-M80032D, ICD-10-D-M80032G, ICD-10-D-M80032K, ICD-10-D-M80032P, ICD-10-D-M80032S, ICD-10-D-M80039A, ICD-10-D-M80039D, ICD-10-D-M80039G, ICD-10-D-M80039K, ICD-10-D-M80039P, ICD-10-D-M80039S, ICD-10-D-M80041A, ICD-10-D-M80041D, ICD-10-D-M80041G, ICD-10-D-M80041K, ICD-10-D-M80041P, ICD-10-D-M80041S, ICD-10-D-M80042A, ICD-10-D-M80042D, ICD-10-D-M80042G, ICD-10-D-M80042K, ICD-10-D-M80042P, ICD-10-D-M80042S, ICD-10-D-M80049A, ICD-10-D-M80049D, ICD-10-D-M80049G, ICD-10-D-M80049K, ICD-10-D-M80049P, ICD-10-D-M80049S, ICD-10-D-M80051A, ICD-10-D-M80051D, ICD-10-D-M80051G, ICD-10-D-M80051K, ICD-10-D-M80051P, ICD-10-D-M80051S, ICD-10-D-M80052A, ICD-10-D-M80052D, ICD-10-D-M80052G, ICD-10-D-M80052K, ICD-10-D-M80052P, ICD-10-D-M80052S, ICD-10-D-M80059A, ICD-10-D-M80059D, ICD-10-D-M80059G, ICD-10-D-M80059K, ICD-10-D-M80059P, ICD-10-D-M80059S, ICD-10-D-M80061A, ICD-10-D-M80061D, ICD-10-D-M80061G, ICD-10-D-M80061K, ICD-10-D-M80061P, ICD-10-D-M80061S, ICD-10-D-M80062A, ICD-10-D-M80062D, ICD-10-D-M80062G, ICD-10-D-M80062K, ICD-10-D-M80062P, ICD-10-D-M80062S, ICD-10-D-M80069A, ICD-10-D-M80069D, ICD-10-D-M80069G, ICD-10-D-M80069K, ICD-10-D-M80069P, ICD-10-D-M80069S, ICD-10-D-M80071A, ICD-10-D-M80071D, ICD-10-D-M80071G, ICD-10-D-M80071K, ICD-10-D-M80071P, ICD-10-D-M80072A, ICD-10-D-M80072D, ICD-10-D-M80072G, ICD-10-D-M80072K, ICD-10-D-M80072P, ICD-10-D-M80072S, ICD-10-D-M80079A, ICD-10-D-M80079D, ICD-10-D-M80079G, ICD-10-D-M80079K, ICD-10-D-M80079P, ICD-10-D-M80079S, ICD-10-D-M8008XA, ICD-10-D-M8008XD, ICD-10-D-M8008XG, ICD-10-D-M8008XK, ICD-10-D-M8008XP, ICD-10-D-M8008XS, ICD-10-D-M800AXA, ICD-10-D-M800AXD, ICD-10-D-M800AXG, ICD-10-D-M800AXK, ICD-10-D-M800AXP, ICD-10-D-M800AXS, ICD-10-D-M8080XA, ICD-10-D-M8080XD, ICD-10-D-M8080XG, ICD-10-D-M8080XK, ICD-10-D-M8080XP, ICD-10-D-M8080XS, ICD-10-D-M80811A, ICD-10-D-M80811D, ICD-10-D-M80811G, ICD-10-D-M80811K, ICD-10-D-M80811P, ICD-10-D-M80811S, ICD-10-D-M80812A, ICD-10-D-M80812D, ICD-10-D-M80812G, ICD-10-D-M80812K, ICD-10-D-M80812P, ICD-10-D-M80812S, ICD-10-D-M80819A, ICD-10-D-M80819D, ICD-10-D-M80819G, ICD-10-D-M80819P, ICD-10-D-M80819S, ICD-10-D-M80821A, ICD-10-D-M80821D, ICD-10-D-M80821G, ICD-10-D-M80821K, ICD-10-D-M80821P, ICD-10-D-M80821S, ICD-10-D-M80822A, ICD-10-D-M80822D, ICD-10-D-M80822G, ICD-10-D-M80822K, ICD-10-D-M80822P, ICD-10-D-M80822S, ICD-10-D-M80829A, ICD-10-D-M80829D, ICD-10-D-M80829G, ICD-10-D-M80829K, ICD-10-D-M80829P, ICD-10-D-M80829S, ICD-10-D-M80831A, ICD-10-D-M80831D, ICD-10-D-M80831G, ICD-10-D-M80831K, ICD-10-D-M80831P, ICD-10-D-M80831S, ICD-10-D-M80832A, ICD-10-D-M80832D, ICD-10-D-M80832G, ICD-10-D-M80832K, ICD-10-D-M80832P, ICD-10-D-M80832S, ICD-10-D-M80839A, ICD-10-D-M80839D, ICD-10-D-M80839G, ICD-10-D-M80839K, ICD-10-D-M80839P, ICD-10-D-M80839S, ICD-10-D-M80841A, ICD-10-D-M80841D, ICD-10-D-M80841G, ICD-10-D-M80841K, ICD-10-D-M80841P, ICD-10-D-M80841S, ICD-10-D-M80842A, ICD-10-D-M80842D, ICD-10-D-M80842G, ICD-10-D-M80842K, ICD-10-D-M80842P, ICD-10-D-M80842S, ICD-10-D-M80849A, ICD-10-D-M80849D, ICD-10-D-M80849G, ICD-10-D-M80849K, ICD-10-D-M80849P, ICD-10-D-M80849S, ICD-10-D-M80851A, ICD-10-D-M80851D, ICD-10-D-M80851G, ICD-10-D-M80851K, ICD-10-D-M80851P, ICD-10-D-M80851S, ICD-10-D-M80852A, ICD-10-D-M80852D, ICD-10-D-M80852G, ICD-10-D-M80852K, ICD-10-D-M80852P, ICD-10-D-M80852S, ICD-10-D-M80859A, ICD-10-D-M80859D, ICD-10-D-M80859G, ICD-10-D-M80859K, ICD-10-D-M80859P, ICD-10-D-M80859S, ICD-10-D-M80861A, ICD-10-D-M80861D, ICD-10-D-M80861G, ICD-10-D-M80861K, ICD-10-D-M80861P, ICD-10-D-M80861S, ICD-10-D-M80862A, ICD-10-D-M80862D, ICD-10-D-M80862G, ICD-10-D-M80862K, ICD-10-D-M80862P, ICD-10-D-M80862S, ICD-10-D-M80869A, ICD-10-D-M80869D, ICD-10-D-M80869G, ICD-10-D-M80869K, ICD-10-D-M80869P, ICD-10-D-M80869S, ICD-10-D-M80871A, ICD-10-D-M80871D, ICD-10-D-M80871G, ICD-10-D-M80871K, ICD-10-D-M80871P, ICD-10-D-M80871S, ICD-10-D-M80872A, ICD-10-D-M80872D, ICD-10-D-M80872G, ICD-10-D-M80872K, ICD-10-D-M80872P, ICD-10-D-M80872S, ICD-10-D-M80879A, ICD-10-D-M80879D, ICD-10-D-M80879G, ICD-10-D-M80879K, ICD-10-D-M80879P, ICD-10-D-M80879S, ICD-10-D-M8088XA, ICD-10-D-M8088XD, ICD-10-D-M8088XG, ICD-10-D-M8088XK, ICD-10-D-M8088XP, ICD-10-D-M8088XS, ICD-10-D-M808AXA, ICD-10-D-M808AXD, ICD-10-D-M808AXG, ICD-10-D-M808AXK, ICD-10-D-M808AXP, ICD-10-D-M808AXS
Bipolar disorder (all codes)	ICD-10-D-F310, ICD-10-D-F3110, ICD-10-D-F3111, ICD-10-D-F3112, ICD-10-D-F3113, ICD-10-D-F312, ICD-10-D-F3130, ICD-10-D-F3131, ICD-10-D-F3132, ICD-10-D-F314, ICD-10-D-F315, ICD-10-D-F3160, ICD-10-D-F3161, ICD-10-D-F3162, ICD-10-D-F3163, ICD-10-D-F3164, ICD-10-D-F3170, ICD-10-D-F3171, ICD-10-D-F3172, ICD-10-D-F3173, ICD-10-D-F3174, ICD-10-D-F3175, ICD-10-D-F3176, ICD-10-D-F3177, ICD-10-D-F3178, ICD-10-D-F3181, ICD-10-D-F3189, ICD-10-D-F319, ICD-9-D-29600, ICD-9-D-29601, ICD-9-D-29602, ICD-9-D-29603, ICD-9-D-29604, ICD-9-D-29605, ICD-9-D-29606, ICD-9-D-29640, ICD-9-D-29641, ICD-9-D-29642, ICD-9-D-29643, ICD-9-D-29644, ICD-9-D-29645, ICD-9-D-29646, ICD-9-D-29650, ICD-9-D-29651, ICD-9-D-29652, ICD-9-D-29653, ICD-9-D-29654, ICD-9-D-29655, ICD-9-D-29656, ICD-9-D-29660, ICD-9-D-29661, ICD-9-D-29662, ICD-9-D-29663, ICD-9-D-29664, ICD-9-D-29665, ICD-9-D-29666, ICD-9-D-2967, ICD-9-D-29680, ICD-9-D-29689
Bipolar disorder – mild	ICD-10-D-F3111, ICD-10-D-F3131, ICD-10-D-F3161, ICD-9-D-29601, ICD-9-D-29641, ICD-9-D-29651, ICD-9-D-29661
Bipolar disorder – moderate	ICD-10-D-F3112, ICD-10-D-F3132, ICD-10-D-F3162, ICD-9-D-29602, ICD-9-D-29642, ICD-9-D-29652, ICD-9-D-29662
Bipolar disorder – severe	ICD-10-D-F3113, ICD-10-D-F312, ICD-10-D-F314, ICD-10-D-F315, ICD-10-D-F3163, ICD-10-D-F3164, ICD-9-D-29603, ICD-9-D-29604, ICD-9-D-29643, ICD-9-D-29644, ICD-9-D-29653, ICD-9-D-29654, ICD-9-D-29663, ICD-9-D-29664
Lithium	DRUG-LITHIUM, DRUG-LITHIUM_CARBONATE, DRUG-LITHIUM_CARBONATE_ER, DRUG-LITHIUM_CITRATE, DRUG-LITHIUM_CITRATE_TETRAHYDRATE, GENERIC_DRUG-LITHIUM_ASPARTATE, GENERIC_DRUG-LITHIUM_CARBONATE, GENERIC_DRUG-LITHIUM_CITRATE, GENERIC_DRUG-LITHIUM_CITRATE_TETRAHYDRATE, NDC-00054002025, NDC-00054002125, NDC-00054002129, NDC-00054252625, NDC-00054252725, NDC-00054252731, NDC-00054253125, NDC-00054352763, NDC-00054452725, NDC-00054452731, NDC-00054852625, NDC-00054852725, NDC-00054852825, NDC-00054852904, NDC-00054853125, NDC-00143127701, NDC-00143127709, NDC-00143127730, NDC-00143130001, NDC-00143130010, NDC-00143318801, NDC-00143318901, NDC-00143318910, NDC-00143319001, NDC-00182178101, NDC-00182178110, NDC-00247017914, NDC-00378130001, NDC-00378145001, NDC-00555034502, NDC-00603422021, NDC-00665416009, NDC-00904291416, NDC-00904564960, NDC-00904613660, NDC-00904613661, NDC-00904613680, NDC-00904620460, NDC-00904620560, NDC-16590078190, NDC-31722054401, NDC-31722054501, NDC-31722054510, NDC-31722054601, NDC-43063019660, NDC-49452408502, NDC-50962052510, NDC-51079014220, NDC-51079018020, NDC-51079026901, NDC-51079026920, NDC-51079065310, NDC-51927312600, NDC-53265027011, NDC-53265028310, NDC-54868133500, NDC-54868133505, NDC-54868363200, NDC-54868523901, NDC-58864066842, NDC-58864081960, NDC-58864082160, NDC-60429026701, NDC-60432061616, NDC-60505250401, NDC-60505250402, NDC-60505250403, NDC-62559028001, NDC-62756043018, NDC-62756043088, NDC-63739026501, NDC-63739026510, NDC-67544053470, NDC-68084004301, NDC-68084054801, NDC-68084064001, NDC-68084065501, NDC-68094075762, NDC-68094076759, NDC-68094076762, NDC-68115070190, NDC-68462022001, NDC-68462022101, NDC-68462022110, NDC-68462022201, NDC-68462022301, NDC-68462022401, NDC-68968449201
THA Component Revision	CPT-27134, CPT-27137, CPT-27138
THA PJI	ICD-9-D-99666, ICD-10-D-T8450XA, ICD-10-D-T8451XA, ICD-10-D-T8452XA
THA Periprosthetic Fracture	ICD-10-D-M9702XA, ICD-10-D-M9701XA, ICD-9-D-99644
THA Aseptic Loosening	ICD-10-D-T84021A, ICD-10-D-T84020A, ICD-9-D-99642
Nonunion	ICD-10-D-S32401K, ICD-10-D-S32402K, ICD-10-D-S32409K, ICD-10-D-S32411K, ICD-10-D-S32412K, ICD-10-D-S32413K, ICD-10-D-S32414K, ICD-10-D-S32415K, ICD-10-D-S32416K, ICD-10-D-S32421K, ICD-10-D-S32422K, ICD-10-D-S32423K, ICD-10-D-S32424K, ICD-10-D-S32425K, ICD-10-D-S32426K, ICD-10-D-S32431K, ICD-10-D-S32432K, ICD-10-D-S32433K, ICD-10-D-S32434K, ICD-10-D-S32435K, ICD-10-D-S32436K, ICD-10-D-S32441K, ICD-10-D-S32442K, ICD-10-D-S32443K, ICD-10-D-S32444K, ICD-10-D-S32445K, ICD-10-D-S32446K, ICD-10-D-S32451K, ICD-10-D-S32452K, ICD-10-D-S32453K, ICD-10-D-S32454K, ICD-10-D-S32455K, ICD-10-D-S32456K, ICD-10-D-S32461K, ICD-10-D-S32462K, ICD-10-D-S32463K, ICD-10-D-S32464K, ICD-10-D-S32465K, ICD-10-D-S32466K, ICD-10-D-S32471K, ICD-10-D-S32472K, ICD-10-D-S32473K, ICD-10-D-S32474K, ICD-10-D-S32475K, ICD-10-D-S32476K, ICD-10-D-S32481K, ICD-10-D-S32482K, ICD-10-D-S32483K, ICD-10-D-S32484K, ICD-10-D-S32485K, ICD-10-D-S32486K, ICD-10-D-S32491K, ICD-10-D-S32492K, ICD-10-D-S32499K, ICD-10-D-S32501K, ICD-10-D-S32502K, ICD-10-D-S32509K, ICD-10-D-S32511K, ICD-10-D-S32512K, ICD-10-D-S32519K, ICD-10-D-S32591K, ICD-10-D-S32592K, ICD-10-D-S32599K, ICD-10-D-S32601K, ICD-10-D-S32602K, ICD-10-D-S32609K, ICD-10-D-S32611K, ICD-10-D-S32612K, ICD-10-D-S32613K, ICD-10-D-S32614K, ICD-10-D-S32615K, ICD-10-D-S32691K, ICD-10-D-S32692K, ICD-10-D-S32699K, ICD-10-D-S32810K, ICD-10-D-S32811K, ICD-10-D-S3282XK, ICD-10-D-S3289XK, ICD-10-D-S72001K, ICD-10-D-S72001M, ICD-10-D-S72001N, ICD-10-D-S72002K, ICD-10-D-S72002M, ICD-10-D-S72002N, ICD-10-D-S72009K, ICD-10-D-S72009M, ICD-10-D-S72009N, ICD-10-D-S72011K, ICD-10-D-S72011M, ICD-10-D-S72011N, ICD-10-D-S72012K, ICD-10-D-S72012M, ICD-10-D-S72012N, ICD-10-D-S72019K, ICD-10-D-S72019M, ICD-10-D-S72019N, ICD-10-D-S72021K, ICD-10-D-S72021M, ICD-10-D-S72021N, ICD-10-D-S72022K, ICD-10-D-S72022M, ICD-10-D-S72022N, ICD-10-D-S72023K, ICD-10-D-S72024K, ICD-10-D-S72024M, ICD-10-D-S72024N, ICD-10-D-S72025K, ICD-10-D-S72025M, ICD-10-D-S72025N, ICD-10-D-S72026K, ICD-10-D-S72031K, ICD-10-D-S72031N, ICD-10-D-S72032K, ICD-10-D-S72032M, ICD-10-D-S72032N, ICD-10-D-S72033K, ICD-10-D-S72033N, ICD-10-D-S72034K, ICD-10-D-S72034M, ICD-10-D-S72034N, ICD-10-D-S72035K, ICD-10-D-S72035N, ICD-10-D-S72036K, ICD-10-D-S72041K, ICD-10-D-S72041M, ICD-10-D-S72041N, ICD-10-D-S72042K, ICD-10-D-S72042M, ICD-10-D-S72042N, ICD-10-D-S72043K, ICD-10-D-S72043N, ICD-10-D-S72044K, ICD-10-D-S72044M, ICD-10-D-S72044N, ICD-10-D-S72045K, ICD-10-D-S72045M, ICD-10-D-S72045N, ICD-10-D-S72046K, ICD-10-D-S72046M, ICD-10-D-S72051K, ICD-10-D-S72051M, ICD-10-D-S72051N, ICD-10-D-S72052K, ICD-10-D-S72052M, ICD-10-D-S72052N, ICD-10-D-S72059K, ICD-10-D-S72059M, ICD-10-D-S72061K, ICD-10-D-S72061N, ICD-10-D-S72062K, ICD-10-D-S72062M, ICD-10-D-S72062N, ICD-10-D-S72063K, ICD-10-D-S72063N, ICD-10-D-S72064K, ICD-10-D-S72064M, ICD-10-D-S72065K, ICD-10-D-S72065M, ICD-10-D-S72065N, ICD-10-D-S72066K, ICD-10-D-S72091K, ICD-10-D-S72091M, ICD-10-D-S72091N, ICD-10-D-S72092K, ICD-10-D-S72092M, ICD-10-D-S72092N, ICD-10-D-S72099K, ICD-10-D-S72099M, ICD-10-D-S72099N, ICD-10-D-S72101K, ICD-10-D-S72101M, ICD-10-D-S72101N, ICD-10-D-S72102K, ICD-10-D-S72102M, ICD-10-D-S72102N, ICD-10-D-S72109K, ICD-10-D-S72109M, ICD-10-D-S72109N, ICD-10-D-S72111K, ICD-10-D-S72111M, ICD-10-D-S72111N, ICD-10-D-S72112K, ICD-10-D-S72112M, ICD-10-D-S72112N, ICD-10-D-S72113K, ICD-10-D-S72113M, ICD-10-D-S72113N, ICD-10-D-S72114K, ICD-10-D-S72114M, ICD-10-D-S72114N, ICD-10-D-S72115K, ICD-10-D-S72115M, ICD-10-D-S72115N, ICD-10-D-S72116K, ICD-10-D-S72116M, ICD-10-D-S72121K, ICD-10-D-S72121M, ICD-10-D-S72121N, ICD-10-D-S72122K, ICD-10-D-S72122M, ICD-10-D-S72122N, ICD-10-D-S72123K, ICD-10-D-S72123M, ICD-10-D-S72124K, ICD-10-D-S72125K, ICD-10-D-S72125M, ICD-10-D-S72131K, ICD-10-D-S72131M, ICD-10-D-S72131N, ICD-10-D-S72132K, ICD-10-D-S72132M, ICD-10-D-S72132N, ICD-10-D-S72133K, ICD-10-D-S72133M, ICD-10-D-S72134K, ICD-10-D-S72135K, ICD-10-D-S72135M, ICD-10-D-S72135N, ICD-10-D-S72136K, ICD-10-D-S72141K, ICD-10-D-S72141M, ICD-10-D-S72141N, ICD-10-D-S72142K, ICD-10-D-S72142M, ICD-10-D-S72142N, ICD-10-D-S72143K, ICD-10-D-S72143M, ICD-10-D-S72143N, ICD-10-D-S72144K, ICD-10-D-S72144M, ICD-10-D-S72144N, ICD-10-D-S72145K, ICD-10-D-S72145M, ICD-10-D-S72145N, ICD-10-D-S72146K, ICD-10-D-S72146M, ICD-10-D-S72146N, ICD-10-D-S7221XK, ICD-10-D-S7221XM, ICD-10-D-S7221XN, ICD-10-D-S7222XK, ICD-10-D-S7222XM, ICD-10-D-S7222XN, ICD-10-D-S7223XK, ICD-10-D-S7223XM, ICD-10-D-S7223XN, ICD-10-D-S7224XK, ICD-10-D-S7224XN, ICD-10-D-S7225XK, ICD-10-D-S7225XM, ICD-10-D-S7225XN, ICD-10-D-S7226XK, ICD-10-D-S7226XM, ICD-10-D-S72301K, ICD-10-D-S72301M, ICD-10-D-S72301N, ICD-10-D-S72302K, ICD-10-D-S72302M, ICD-10-D-S72302N, ICD-10-D-S72309K, ICD-10-D-S72309M, ICD-10-D-S72309N, ICD-10-D-S72321K, ICD-10-D-S72321M, ICD-10-D-S72321N, ICD-10-D-S72322K, ICD-10-D-S72322M, ICD-10-D-S72322N, ICD-10-D-S72323K, ICD-10-D-S72323M, ICD-10-D-S72323N, ICD-10-D-S72324K, ICD-10-D-S72324M, ICD-10-D-S72324N, ICD-10-D-S72325K, ICD-10-D-S72325M, ICD-10-D-S72325N, ICD-10-D-S72326K, ICD-10-D-S72331K, ICD-10-D-S72331M, ICD-10-D-S72331N, ICD-10-D-S72332K, ICD-10-D-S72332M, ICD-10-D-S72332N, ICD-10-D-S72333K, ICD-10-D-S72333M, ICD-10-D-S72333N, ICD-10-D-S72334K, ICD-10-D-S72334N, ICD-10-D-S72335K, ICD-10-D-S72335M, ICD-10-D-S72336K, ICD-10-D-S72341K, ICD-10-D-S72341M, ICD-10-D-S72342K, ICD-10-D-S72342M, ICD-10-D-S72342N, ICD-10-D-S72343K, ICD-10-D-S72343M, ICD-10-D-S72344K, ICD-10-D-S72344M, ICD-10-D-S72344N, ICD-10-D-S72345K, ICD-10-D-S72345M, ICD-10-D-S72346K, ICD-10-D-S72351K, ICD-10-D-S72351M, ICD-10-D-S72351N, ICD-10-D-S72352K, ICD-10-D-S72352M, ICD-10-D-S72352N, ICD-10-D-S72353K, ICD-10-D-S72353M, ICD-10-D-S72353N, ICD-10-D-S72354K, ICD-10-D-S72354M, ICD-10-D-S72354N, ICD-10-D-S72355K, ICD-10-D-S72355M, ICD-10-D-S72355N, ICD-10-D-S72356K, ICD-10-D-S72361K, ICD-10-D-S72361M, ICD-10-D-S72361N, ICD-10-D-S72362K, ICD-10-D-S72362M, ICD-10-D-S72362N, ICD-10-D-S72363K, ICD-10-D-S72363N, ICD-10-D-S72364K, ICD-10-D-S72364M, ICD-10-D-S72364N, ICD-10-D-S72365K, ICD-10-D-S72365M, ICD-10-D-S72366K, ICD-10-D-S72366M, ICD-10-D-S72391K, ICD-10-D-S72391M, ICD-10-D-S72391N, ICD-10-D-S72392K, ICD-10-D-S72392M, ICD-10-D-S72392N, ICD-10-D-S72399K, ICD-10-D-S72399N, ICD-9-D-73382
Delayed union	ICD-10-D-S32401G, ICD-10-D-S32402G, ICD-10-D-S32409G, ICD-10-D-S32411G, ICD-10-D-S32412G, ICD-10-D-S32413G, ICD-10-D-S32414G, ICD-10-D-S32415G, ICD-10-D-S32421G, ICD-10-D-S32422G, ICD-10-D-S32423G, ICD-10-D-S32424G, ICD-10-D-S32425G, ICD-10-D-S32426G, ICD-10-D-S32431G, ICD-10-D-S32432G, ICD-10-D-S32433G, ICD-10-D-S32434G, ICD-10-D-S32435G, ICD-10-D-S32436G, ICD-10-D-S32441G, ICD-10-D-S32442G, ICD-10-D-S32443G, ICD-10-D-S32444G, ICD-10-D-S32445G, ICD-10-D-S32446G, ICD-10-D-S32451G, ICD-10-D-S32452G, ICD-10-D-S32454G, ICD-10-D-S32455G, ICD-10-D-S32456G, ICD-10-D-S32461G, ICD-10-D-S32462G, ICD-10-D-S32463G, ICD-10-D-S32464G, ICD-10-D-S32465G, ICD-10-D-S32466G, ICD-10-D-S32471G, ICD-10-D-S32472G, ICD-10-D-S32473G, ICD-10-D-S32474G, ICD-10-D-S32475G, ICD-10-D-S32481G, ICD-10-D-S32482G, ICD-10-D-S32483G, ICD-10-D-S32484G, ICD-10-D-S32485G, ICD-10-D-S32486G, ICD-10-D-S32491G, ICD-10-D-S32492G, ICD-10-D-S32499G,ICD-10-D-M80051G, ICD-10-D-M80052G, ICD-10-D-M80059G, ICD-10-D-M80851G, ICD-10-D-M80852G, ICD-10-D-M80859G, ICD-10-D-M84351G, ICD-10-D-M84352G, ICD-10-D-M84353G, ICD-10-D-M84451G, ICD-10-D-M84452G, ICD-10-D-M84453G, ICD-10-D-M84551G, ICD-10-D-M84552G, ICD-10-D-M84553G, ICD-10-D-M84651G, ICD-10-D-M84652G, ICD-10-D-M84653G, ICD-10-D-S72101G, ICD-10-D-S72101H, ICD-10-D-S72101J, ICD-10-D-S72102G, ICD-10-D-S72102H, ICD-10-D-S72102J, ICD-10-D-S72109G, ICD-10-D-S72109H, ICD-10-D-S72111G, ICD-10-D-S72111H, ICD-10-D-S72111J, ICD-10-D-S72112G, ICD-10-D-S72112H, ICD-10-D-S72112J, ICD-10-D-S72113G, ICD-10-D-S72113H, ICD-10-D-S72113J, ICD-10-D-S72114G, ICD-10-D-S72114H, ICD-10-D-S72114J, ICD-10-D-S72115G, ICD-10-D-S72115H, ICD-10-D-S72115J, ICD-10-D-S72116G, ICD-10-D-S72116J, ICD-10-D-S72121G, ICD-10-D-S72121H, ICD-10-D-S72122G, ICD-10-D-S72122H, ICD-10-D-S72122J, ICD-10-D-S72123G, ICD-10-D-S72124G, ICD-10-D-S72125G, ICD-10-D-S72125H, ICD-10-D-S72125J, ICD-10-D-S72126G, ICD-10-D-S72141G, ICD-10-D-S72141H, ICD-10-D-S72141J, ICD-10-D-S72142G, ICD-10-D-S72142H, ICD-10-D-S72142J, ICD-10-D-S72143G, ICD-10-D-S72143H, ICD-10-D-S72143J, ICD-10-D-S72144G, ICD-10-D-S72144H, ICD-10-D-S72144J, ICD-10-D-S72145G, ICD-10-D-S72145H, ICD-10-D-S72145J, ICD-10-D-S72146G, ICD-10-D-S72146H, ICD-10-D-S72146J, ICD-10-D-S7221XG, ICD-10-D-S7221XH, ICD-10-D-S7221XJ, ICD-10-D-S7222XG, ICD-10-D-S7222XH, ICD-10-D-S7222XJ, ICD-10-D-S7223XG, ICD-10-D-S7223XH, ICD-10-D-S7223XJ, ICD-10-D-S7224XG, ICD-10-D-S7224XH, ICD-10-D-S7225XG, ICD-10-D-S7225XH, ICD-10-D-S7225XJ, ICD-10-D-S7226XG, ICD-10-D-S7226XH, ICD-10-D-S7226XJ, ICD-10-D-S72301G, ICD-10-D-S72301H, ICD-10-D-S72301J, ICD-10-D-S72302G, ICD-10-D-S72302H, ICD-10-D-S72302J, ICD-10-D-S72309G, ICD-10-D-S72309H, ICD-10-D-S72309J, ICD-10-D-S72321G, ICD-10-D-S72321H, ICD-10-D-S72321J, ICD-10-D-S72322G, ICD-10-D-S72322H, ICD-10-D-S72322J, ICD-10-D-S72323G, ICD-10-D-S72324G, ICD-10-D-S72324H, ICD-10-D-S72325G, ICD-10-D-S72325H, ICD-10-D-S72325J, ICD-10-D-S72326G, ICD-10-D-S72326H, ICD-10-D-S72331G, ICD-10-D-S72331H, ICD-10-D-S72331J, ICD-10-D-S72332G, ICD-10-D-S72332H, ICD-10-D-S72332J, ICD-10-D-S72333G, ICD-10-D-S72333J, ICD-10-D-S72334G, ICD-10-D-S72334H, ICD-10-D-S72335G, ICD-10-D-S72335H, ICD-10-D-S72335J, ICD-10-D-S72336G, ICD-10-D-S72341G, ICD-10-D-S72341H, ICD-10-D-S72341J, ICD-10-D-S72342G, ICD-10-D-S72342H, ICD-10-D-S72342J, ICD-10-D-S72343G, ICD-10-D-S72344G, ICD-10-D-S72344H, ICD-10-D-S72345G, ICD-10-D-S72346G, ICD-10-D-S72351G, ICD-10-D-S72351H, ICD-10-D-S72351J, ICD-10-D-S72352G, ICD-10-D-S72352H, ICD-10-D-S72352J, ICD-10-D-S72353G, ICD-10-D-S72353H, ICD-10-D-S72353J, ICD-10-D-S72354G, ICD-10-D-S72354H, ICD-10-D-S72354J, ICD-10-D-S72355G, ICD-10-D-S72355H, ICD-10-D-S72355J, ICD-10-D-S72356G, ICD-10-D-S72361G, ICD-10-D-S72361H, ICD-10-D-S72361J, ICD-10-D-S72362G, ICD-10-D-S72362H, ICD-10-D-S72362J, ICD-10-D-S72363G, ICD-10-D-S72363J, ICD-10-D-S72364G, ICD-10-D-S72364H, ICD-10-D-S72364J, ICD-10-D-S72365G, ICD-10-D-S72365H, ICD-10-D-S72365J, ICD-10-D-S72366G, ICD-10-D-S72391G, ICD-10-D-S72391H, ICD-10-D-S72391J, ICD-10-D-S72392G, ICD-10-D-S72392H, ICD-10-D-S72392J, ICD-10-D-S72399G, ICD-10-D-S72401G, ICD-10-D-S72401H, ICD-10-D-S72401J, ICD-10-D-S72402G, ICD-10-D-S72402H, ICD-10-D-S72402J, ICD-10-D-S72409G, ICD-10-D-S72409H, ICD-10-D-S728X1G, ICD-10-D-S728 × 1H, ICD-10-D-S728 × 1J, ICD-10-D-S728X2G, ICD-10-D-S728 × 2H, ICD-10-D-S728 × 2J, ICD-10-D-S728X9G, ICD-10-D-S728 × 9H, ICD-10-D-S728 × 9J, ICD-10-D-S7290XG, ICD-10-D-S7290XH, ICD-10-D-S7290XJ, ICD-10-D-S7291XG, ICD-10-D-S7291XH, ICD-10-D-S7291XJ, ICD-10-D-S7292XG, ICD-10-D-S7292XH, ICD-10-D-S7292XJ, ICD-10-D-S32401G, ICD-10-D-S32402G, ICD-10-D-S32409G, ICD-10-D-S32411G, ICD-10-D-S32412G, ICD-10-D-S32413G, ICD-10-D-S32414G, ICD-10-D-S32415G, ICD-10-D-S32421G, ICD-10-D-S32422G, ICD-10-D-S32423G, ICD-10-D-S32424G, ICD-10-D-S32425G, ICD-10-D-S32426G, ICD-10-D-S32431G, ICD-10-D-S32432G, ICD-10-D-S32433G, ICD-10-D-S32434G, ICD-10-D-S32435G, ICD-10-D-S32436G, ICD-10-D-S32441G, ICD-10-D-S32442G, ICD-10-D-S32443G, ICD-10-D-S32444G, ICD-10-D-S32445G, ICD-10-D-S32446G, ICD-10-D-S32451G, ICD-10-D-S32452G, ICD-10-D-S32454G, ICD-10-D-S32455G, ICD-10-D-S32456G, ICD-10-D-S32461G, ICD-10-D-S32462G, ICD-10-D-S32463G, ICD-10-D-S32464G, ICD-10-D-S32465G, ICD-10-D-S32466G, ICD-10-D-S32471G, ICD-10-D-S32472G, ICD-10-D-S32473G, ICD-10-D-S32474G, ICD-10-D-S32475G, ICD-10-D-S32481G, ICD-10-D-S32482G, ICD-10-D-S32483G, ICD-10-D-S32484G, ICD-10-D-S32485G, ICD-10-D-S32486G, ICD-10-D-S32491G, ICD-10-D-S32492G, ICD-10-D-S32499G

CPT, Current Procedural Terminology.

**Table A.2:** Sensitivity Analysis Comparing Medical and Surgical Complication Rates among THA Patients with No Lithium Use versus those on Lithium with Additional Matching based on Bipolar Disease Severity^a^

90 D postoperative	Total (n = 4614)	No lithium use (n = 3681)	Lithium use (n = 933)	*P* value
Readmissions	403 (8.7%)	343 (9.3%)	60 (6.4%)	**<.01**
Any medical complication	462 (10%)	379 (10.3%)	83 (8.9%)	.23
Acute kidney injury	85 (1.8%)	72 (2%)	13 (1.4%)	.32
Cardiac arrest	<11 (<0.2%)	<11 (<0.3%)	0 (0.0%)	.57
Deep vein thrombosis	34 (0.7%)	27 (0.7%)	<11 (<1.2%)	1.00
Wound dehiscence	61 (1.3%)	51 (1.4%)	<11 (<1.2%)	.56
Hematoma	36 (0.8%)	32 (0.9%)	<11 (<1.2%)	.25
Nerve injury	0 (0.0%)	0 (0.0%)	0 (0.0%)	-
Pneumonia	59 (1.3%)	54 (1.5%)	<11 (<1.2%)	.04
Pulmonary embolism	13 (0.3%)	13 (0.4%)	0 (0.0%)	.14
Transfusion	108 (2.3%)	91 (2.5%)	17 (1.8%)	.29
Urinary tract infection	168 (3.6%)	133 (3.6%)	35 (3.8%)	.92
Periprosthetic fracture	64 (1.4%)	52 (1.4%)	12 (1.3%)	.89
Aseptic loosening	<11 (<0.2%)	<11 (<0.3%)	<11 (<1.2%)	1.00
Revision surgery	93 (2.0%)	75 (2.0%)	18 (1.9%)	.94
PJI	98 (2.1%)	85 (2.3%)	13 (1.4%)	.09

2 Y postoperative	Total (n = 3692)	No lithium use (n = 2942)	Lithium use (n = 750)	*P* value
Periprosthetic fracture	73 (2.0%)	61 (2.1%)	12 (1.6%)	.49
Aseptic loosening	58 (1.6%)	47 (1.6%)	11 (1.5%)	.93
Revision surgery	148 (4.0%)	118 (4.0%)	30 (4.0%)	1.00
PJI	142 (3.8%)	122 (4.1%)	20 (2.7%)	.08

Bold values indicate *P* values which meet the significance threshold of α = 0.05.

a Propensity-score matched between nonlithium users and lithium-use patients in 4:1 ratio based on age, diagnosis of bipolar disorder, bipolar disease severity, gender, CCI, body mass index, and insurance type.

## Appendix A Supplementary data

Conflict of Interest Statement for McLawhorn

Conflict of Interest Statement for Basseri

Conflict of Interest Statement for Kim

Conflict of Interest Statement for Mazzucco

Conflict of Interest Statement for Rahman

## References

[1] Abdel M.P., Cottino U., Mabry T.M. (2015). Management of periprosthetic femoral fractures following total hip arthroplasty: a review. Int Orthop.

[2] Moreta J., Uriarte I., Bidea I., Foruria X., Legarreta M.J., Etxebarría-Foronda I. (2021). High mortality rate following periprosthetic femoral fractures after total hip arthroplasty. A multicenter retrospective study. Injury.

[3] Binkley N., Nickel B., Anderson P.A. (2023). Periprosthetic fractures: an unrecognized osteoporosis crisis. Osteoporos Int.

[4] Russell L.A. (2013). Osteoporosis and orthopedic surgery: effect of bone health on total joint arthroplasty outcome. Curr Rheumatol Rep.

[5] Nixon M., Taylor G., Sheldon P., Iqbal S.J., Harper W. (2007). Does bone quality predict loosening of cemented total hip replacements?. J Bone Joint Surg Br.

[6] Zhu Y., Chen W., Sun T., Zhang X., Liu S., Zhang Y. (2015). Risk factors for the periprosthetic fracture after total hip arthroplasty: a systematic review and meta-analysis. Scand J Surg.

[7] Bernatz J.T., Brooks A.E., Squire M.W., Illgen R.I., Binkley N.C., Anderson P.A. (2019). Osteoporosis is common and undertreated prior to total joint arthroplasty. J Arthroplasty.

[8] Holzer L.A., Borotschnig L., Holzer G. (2023). Evaluation of FRAX in patients with periprosthetic fractures following primary total hip and knee arthroplasty. Sci Rep.

[9] Lee J., Kim T., Kim T. (2018). Treatment of periprosthetic femoral fractures following hip arthroplasty. Hip Pelvis.

[10] Chen X., Myers C.A., Clary C.W., Varga P., Coombs D., DeWall R.J. (2022). Impact of bone health on the mechanics of plate fixation for Vancouver B1 periprosthetic femoral fractures. Clin Biomech (Bristol, Avon).

[11] Bansal H. (2022). Medication-related osteonecrosis of the jaw: an update. Natl J Maxillofac Surg.

[12] Beth-Tasdogan N.H., Mayer B., Hussein H., Zolk O., Peter J. (2022). Interventions for managing medication-related osteonecrosis of the jaw. Cochrane Database Syst Rev.

[13] Diker-Cohen T., Rosenberg D., Avni T., Shepshelovich D., Tsvetov G., Gafter-Gvili A. (2020). Risk for infections during treatment with denosumab for osteoporosis: a systematic review and meta-analysis. J Clin Endocrinol Metab.

[14] Drugs.com (2024).

[15] Drugs.com (2024).

[16] Agarwal A.R., Kinnard M.J., Murdock C., Zhao A.Y., Ahiarakwe U., Cohen J.S. (2024). The cost-effectiveness of osteoporosis medications for preventing periprosthetic fractures following femoral neck fracture indicated hip arthroplasty: a break-even analysis. Osteoporos Int.

[17] Cornelissen D., Boonen A., Bours S., Evers S., Dirksen C., Hiligsmann M. (2020). Understanding patients' preferences for osteoporosis treatment: the impact of patients' characteristics on subgroups and latent classes. Osteoporos Int.

[18] Zamani A., Omrani G.R., Nasab M.M. (2009). Lithium's effect on bone mineral density. Bone.

[19] Drugs.com (2024).

[20] Vachhani K., Whyne C., Wang Y., Burns D.M., Nam D. (2018). Low-dose lithium regimen enhances endochondral fracture healing in osteoporotic rodent bone. J Orthop Res.

[21] Wang X., Zhu S., Jiang X., Li Y., Song D., Hu J. (2015). Systemic administration of lithium improves distracted bone regeneration in rats. Calcif Tissue Int.

[22] Jin Y., Xu L., Hu X., Liao S., Pathak J.L., Liu J. (2017). Lithium chloride enhances bone regeneration and implant osseointegration in osteoporotic conditions. J Bone Miner Metab.

[23] Liu B., Wu Q., Zhang S., Del Rosario A. (2019). Lithium use and risk of fracture: a systematic review and meta-analysis of observational studies. Osteoporos Int.

[24] Vestergaard P., Rejnmark L., Mosekilde L. (2005). Reduced relative risk of fractures among users of lithium. Calcif Tissue Int.

[25] Nam D., Balasuberamaniam P., Milner K., Kunz M., Vachhani K., Kiss A. (2020). Lithium for fracture treatment (LiFT): a double-blind randomised control trial protocol. BMJ Open.

[26] Cochrane N.H., Kim B.I., Seyler T.M., Bolognesi M.P., Ryan S.P., Ledford C.K. (2024). Timing of renal transplant prior to total knee arthroplasty impacts 90-Day postoperative outcomes. J Arthroplasty.

[27] Debbi E.M., Mosich G.M., Bendich I., Kapadia M., Ast M.P., Westrich G.H. (2022). Same-day discharge total hip and knee arthroplasty: trends, complications, and readmission rates. J Arthroplasty.

[28] Ng V.W.S., Leung M.T.Y., Lau W.C.Y., Chan E.W., Hayes J.F., Osborn D.P.J. (2024). Lithium and the risk of fractures in patients with bipolar disorder: a population-based cohort study. Psychiatry Res.

[29] Chandrasekaran V., Brennan-Olsen S.L., Stuart A.L., Pasco J.A., Berk M., Hodge J.M. (2019). Bipolar disorder and bone health: a systematic review. J Affect Disord.

[30] Su J., Cheng B., Huang Y., Lee C., Yang Y., Lu M. (2017). Bipolar disorder and the risk of fracture: a nationwide population-based cohort study. J Affect Disord.

[31] Hsu C., Hsu Y., Chang K., Lee C., Chong L., Wang Y. (2016). Increased risk of fracture in patients with bipolar disorder: a nationwide cohort study. Soc Psychiatry Psychiatr Epidemiol.

[32] Lassas A., Norrback K., Adolfsson R., Maripuu M. (2022). Bipolar disorder and bone mineral density Z-Scores in relation to clinical characteristics and lithium medication. J Clin Med.

[33] Li S., Qui Y., Teng Z., Chen J., Kang D., Tang H. (2020). Association between bipolar disorder and low bone mass: a cross-sectional study with newly diagnosed, drug-naïve patients. Front Psychiatry.

[34] Köhler-Forsberg O., Rohde C., Nierenberg A.A., Østergaard S.D. (2022). Association of lithium treatment with the risk of osteoporosis in patients with bipolar disorder. JAMA Psychiatry.

[35] Kim J.H., Liu X., Wang J., Chen X., Zhang H., Kim S.H. (2013). Wnt signaling in bone formation and its therapeutic potential for bone diseases. Ther Adv Musculoskelet Dis.

[36] Vachhani K., Pagotto A., Wang Y., Whyne C., Nam D. (2018). Design of experiments confirms optimization of lithium administration parameters for enhanced fracture healing. J Biomech.

[37] Bernick J., Wang Y., Sigal I.A., Alman B.A., Whyne C.M., Nam D. (2014). Parameters for lithium treatment are critical in its enhancement of fracture-healing in rodents. J Bone Joint Surg Am.

[38] Ruettermann M. (2018). Large databases: additional value or inherent risk?. J Hand Surg Eur Vol.

[39] Talbert S., Sole M.L. (2013). Too much information: research issues associated with large databases. Clin Nurse Spec.

